# The methods and use of questionnaires for the diagnosis of dental phobia by Japanese dental practitioners specializing in special needs dentistry and dental anesthesiology: a cross-sectional study

**DOI:** 10.1186/s12903-022-02071-y

**Published:** 2022-02-11

**Authors:** Mika Ogawa, Terumi Ayuse, Toshiaki Fujisawa, Shuntaro Sato, Takao Ayuse

**Affiliations:** 1 Section of Anesthesiology, Department of Diagnostics and General Care, Fukuoka Dental University, Fukuoka, Japan; 2 Department of Special Care Dentistry, Nagasaki University Hospital, Nagasaki, Japan; 3 Department of Dental Anesthesiology, Hokkaido University, Hokkaido, Japan; 4 Clinical Research Center, Nagasaki University Hospital, Nagasaki, Japan; 5 Department of Dental Anesthesiology, Nagasaki University Institute of Biomedical Sciences, Course of Medical and Dental Sciences, Nagasaki, Japan

**Keywords:** Dental phobia, Questionnaire, Special needs dentistry, Dental anesthesiology, Dental practitioner

## Abstract

**Background:**

Dental phobia is covered by medical insurance; however, the diagnostic methods are not standardized in Japan. Therefore, the aim of this study was to investigate the methods and use of questionnaires for the diagnosis of dental phobia by Japanese dental practitioners specializing in special needs dentistry and dental anesthesiology.

**Methods:**

We conducted an online survey to obtain information from the members of the Japanese Society for Disability and Oral Health (JSDH, n = 5134) and the Japanese Dental Society of Anesthesiology (JDSA, n = 2759). Response items included gender, qualification, affiliation type, methods of diagnosis and management of dental phobia, use of questionnaire, need to establish standardized diagnostic method for dental phobia, and others. The chi-squared test was used to compare answers between the three groups: JSDH only, JDSA only, and both JSDH and JDSA. Multiple logistic regression analysis was conducted to identify factors associated with the use of an assessment questionnaire.

**Results:**

Data were obtained from 614 practitioners (JSDH only, n = 329; JDSA only, n = 195; both JSDH and JDSA: n = 90, response rate: 7.8% [614/7,893], men: n = 364 [58.5%]). Only 9.7% of practitioners used questionnaires to quantify the level of dental anxiety. The members of both JSDH and JDSA group used questionnaires more frequently than members of the JSDH only (19% and 7.1%, respectively; Bonferroni corrected p < 0.01). Most practitioners (89.1%) diagnosed dental phobia based on patient complaints of fear of treatment. Furthermore, majority of the participants (73.3%) felt the need to establish standardized diagnostic method for “dental phobia.” Multiple logistic regression analysis showed that membership of the JSDH only was negatively related (odds ratio [OR] 0.28, 95% confidence interval [CI] 0.13–0.60), and use of behavioral therapy was positively related (OR 2.34, 95% CI 1.18–4.84) to the use of a questionnaire.

**Conclusions:**

The results of this study showed that the use of questionnaires was very low, patients’ subjective opinions were commonly used to diagnose dental phobia, and a standardized diagnostic criterion was thus needed among practitioners. Therefore, it is necessary to establish diagnostic criteria for dental phobia in line with the Japanese clinical system and to educate dentists about them.

## Background

Dental phobia is recognized as extremely high anxiety for dental treatment. In such cases, it is difficult to perform dental treatments using routine methods [1, 2]. Therefore, dental treatments for patients with dental phobia can be performed by utilizing a pharmacological approach, such as cognitive behavioral therapy (CBT) or a psychological approach such as sedation [2, 3]. "Dental phobia" is covered by health insurance in Japan; hence, the term is highly recognized by general dentists. Dental phobia can be accordingly diagnosed in two ways.

The first is a diagnosis according to the Diagnostic and Statistical Manual of Mental Disorders, Fifth Edition (DSM-5). Dental phobia is a specific type of phobia (localized phobia) in the DSM-5 [3]. Specific phobia is a condition in which a person feels fear or anxiety about a specific situation or subject. Diagnosis is based on interviews or self-administered questionnaires formulated according to the DSM-5 criteria. The incidence of dental phobia based on DSM classification is 2.1% (Sweden) [4], 3.1% (USA) [5], and 3.7% (Netherlands) [6]. A diagnosis of dental phobia according to the DSM-5 criteria is often made by doctors or psychologists; however this is not common in Japan.

The second method is to diagnose dental phobia as severe dental anxiety or fear, and to use the cut-off values of the questionnaire. The concept of dental anxiety or fear is a continuum rather than a dichotomy of scary/not scary. Quantification is essential for determining methods of dealing with dental anxiety or fear [7, 8]. Therefore, multiple psychological scales with established reliability and validity have been developed and used worldwide [1–3]. For example, the Modified Dental Anxiety Scale (MDAS) is a five-item questionnaire that quantifies the degree of anxiety in five dental situations [9]. The total score ranges from 5 to 25. An MDAS score of ≥ 19 indicates a high level of dental anxiety and specific phobia [10]. The prevalence of high dental fear (MDAS score ≥ 19) is approximately 10% in Japan [11] and in other countries [9, 12, 13]. However, the concept of dental anxiety is less recognized by Japanese dentists than dental phobia, and the measurement of dental anxiety using questionnaires is not common in Japan.

The treatment of dental phobia varies across countries. In Scandinavian countries, dental phobia is mainly diagnosed by psychologists, and CBT by psychologists is conducted in specialized facilities [14, 15]. Sedation in primary dental care is common in the UK [16]. However, the importance of CBT for dental phobia has also been pointed out, and it has been reported that psychologist-led CBT services are being provided accordingly [17, 18]. In Japan, however, CBT by psychologists is rarely used. Some general dental clinics offer systematic desensitization methods, such as tell-show-do and nitrous oxide inhalation sedation by general dentists. Dental phobia patients who cannot be treated by general dentists are referred to higher medical institutions, such as university hospitals and regional clinics. These institutions often have departments of special-needs dentistry and dental anesthesiology. Experienced dentists are often certified dentists or specialists of the Japanese Society for Disability and Oral Health (JSDH) and the Japanese Dental Society of Anesthesiology (JDSA).

Typical criteria for JSDH specialists include completion of a training program of at least 5 years in total at a prescribed training facility. Opportunities to practice dentistry for the disabled should be routine. JDSA's criteria for specialists include being a member of the JDSA for > 5 years, being in the field of dental anesthesia, and completing the specialist training curriculum. Therefore, the difference in patient demographics between the two organizations is that JSDH specialists typically treat patients with disabilities, while JDSA specialists typically provide general anesthesia, sedation, and pain clinic services for dental treatment and oral surgery. Patients with dental phobia who are referred to higher medical institutions are often treated by JSDH members under sedation or general anesthesia performed by JDSA members [19, 20]. Therefore, it is likely that JSDH and JDSA members have more contact with dental phobia patients than other dentists. However, the measurement of dental anxiety is not included in the training curriculum of the JSDH and JDSA.

The term dental phobia is commonly used among general dentists and even among patients in Japan; however, a clear definition or diagnostic criterion for dental phobia has not been established till date. Therefore, the diagnosis of dental phobia is considered to depend on the experience of dentists in Japan. The aim of this study was to investigate the methods and use of questionnaires for the diagnosis of “dental phobia” by Japanese dental practitioners specializing in special needs dentistry and dental anesthesiology. We hypothesized that the diagnosis of dental phobia in our participants would be subjective, that the use of questionnaires would be low, and that they would want to establish standardized diagnostic criteria.

## Methods

Members of the JSDH (total number of members: 5,134) and the JDSA (total number of members: 2759) were targeted for this cross-sectional study. All the methods were performed in accordance with the Declaration of Helsinki. All experimental protocols, including the method of obtaining consent from the participants, were approved by the respective boards of both academic societies (No. 1920-7 from the JDSA and No. 20029 from JSDH). We conducted an online questionnaire survey (total number of requests: 7893) using Google Forms (California, Google LLC). The web address for the questionnaire survey was sent to all members via e-mail newsletters of both academic societies. Participation in the survey was voluntary and anonymous. The aim and other details of the study were explained on the first screen. Clicking on the button located at the bottom of the first screen by the participant was taken as informed consent. The reminder was given twice via e-mail newsletters to the participants. Anonymous responses between August 7, 2020, and August 22, 2020, were recorded on the Internet.

The questionnaire included the following items:

1. Gender (male, female)
2. Qualification (JSDH: specialist [required years of experiences = 5 years]; certified doctor [3 years]; certified dental hygienist [3 years]; other, JDSA: specialist [5 years]; certified doctor [2 years]; certified dental hygienist [1 year], others [non-certified dentists and hygienist])
3. Affiliation type (individual clinic, university hospital, regional clinic, hospital dentistry)
4. Method for management (behavioral therapy, intravenous sedation, general anesthesia, nitrous oxide inhalation sedation, oral sedation, etc.)
5. Actual diagnostic criteria for dental phobia in new patients (patient complaints of anxiety, experience of feeling unwell during treatment, difficulty in keeping the patient's mouth open during treatment, body movement during treatment, cancellation of dental reception, etc.)
6. Use of a questionnaire or criteria to diagnose dental phobia (yes/no)
7. Dental anxiety assessment indices used (modified Dental Anxiety Scale, Dental Fear Survey, Short version of the Dental Anxiety Inventory, Dental Subscale of Children's Fear Survey Schedule, Visual Analog Scale, State-Trait Anxiety Inventory, State-Trait Anxiety Inventory for Children, and original evaluation criteria)
8. The need to establish standardized diagnostic method for dental phobia (Do you think it is necessary to establish standardized assessment criteria for dental phobia?—very needed, a little needed, either, not needed a little, no need at all) and
9. Percentage of patients with dental phobia treated every week.

### Eligibility criteria

The inclusion criterion was participants who did not respond to their qualification. There are no exclusion criteria.

### Classification of groups

The groupings are defined as follows: members of the “JSDH only” group belong to JSDH but not to JDSA, those of “JDSA only” group belong to JSDA but not to JDSH, and those of “both JSDH and JDSA” group belong to both JSDH and JDSA.

### Statistical analysis

The characteristics and questionnaire answers were summarized as counts and percentages. Fisher’s exact test was used to examine the association between participants’ qualifications and whether they used a questionnaire. The chi-squared test or Fisher’s exact test was used to compare answers between the three groups (JSDH only, JDSA only, and both JSDH and JDSA). The two groups were then compared using the same test, and the P value was corrected for Bonferroni correction. Multiple logistic regression analysis was conducted to identify factors associated with the use of a dental anxiety assessment questionnaire and estimated odds ratios (ORs), 95% confidence intervals (95% CIs), and p-values. This model included gender, membership, facility type, and five types of management as an independent factor. All tests were conducted at a significance level of 0.05. All statistical analyses were performed with R version 4.0.0 (R Foundation for Statistical Computing, Vienna, Austria).

## Results

A total of 7.88% (622/7,893) of the participants responded to the survey. Eight responses had missing qualification data; therefore, 614 responses were finally included in the study. Fifty-eight participants were male (356/614). Almost 10% of the participants (66/614) calculated that the proportion of patients with dental phobia treated every working week was 25% and above. Table 1 shows the relationship between the qualifications of the participants and the use of questionnaires to diagnose dental phobia. Thirteen percent of the JSDH only group members were specialists (53/419), whereas 38.2% of the JDSA only group members were specialists (109/285). There was no statistically significant association between participants’ qualifications and the use of a questionnaire among JSDH only group members (p = 0.97). However, there was a slightly significant association observed between the JDSA only group members (p = 0.049).

**Table 1:** Qualification of the participants and the usage of questionnaire to diagnose dental phobia

		JSDH			JDSA			
		The use of questionnaire n (%)			The use of questionnaire n (%)			
Qualification	Overall n (%)	No	Yes	P	Overall	No	Yes	P
Specialist	53 (12.8)	48 (12.8)	5 (12.5)	0.97	109 (38.2)	99 (39.9)	10 (27.0)	0.049
Certified doctors	193 (46.5)	174 (46.4)	19 (47.5)		132 (46.3)	115 (46.4)	17 (45.9)	
Certified dental hygienist	32 (7.7)	30 (8.0)	2 (5.0)		7 (2.5)	7 (2.8)	0 (0.0)	
Others	137 (33.0)	123 (32.8)	14 (35.0)		37 (13.0)	27 (10.9)	10 (27.0)	
Total	419 (100)	375 (100)	40 (100)		285 (100)	248 (100)	37 (100)	

JSDH = Japanese Society for Disability and Oral Health, JDSA = Japanese Dental Society of Anesthesiology

The common answers and differences among members are shown in Table 2. The percentages of affiliated facility type, use of questionnaires, actual diagnostic criteria, need for standardized diagnostic methods, and methods of management were significantly different between the groups. Approximately one-third of the respondents worked at an individual clinic, and one-third worked at a university hospital. Behavioral therapy was significantly used more frequently among members of the JSDH only (JSDH only group, 264/329 [80%]) than among members of the JDSA only (JDSA only group, 66/195 [34%], corrected p < 0.01). Intravenous sedation was significantly more common in the JDSA only group than in the JSDH only group (170/195 [87%] and 162/329 [49%], respectively; corrected p < 0.01).

**Table 2:** The difference on the answer between memberships

	Overall	JSDH only	JDSA only	Both JSDH and JDSA	P*
	N = 614	N = 329	N = 195	N = 90	
	n (%)	n (%)	n (%)	n (%)	
**Gender**					0.074
Male	356 (58)	197 (61)	101 (52)	58 (64)	
Female	253 (42)	128 (39)	93 (48)	32 (36)	
(Missing)	5	4	1	0	
**Affiliation type**					0.041
Individual clinic	223 (36)	122 (37)	73 (37)	28 (31)	
University hospital	194 (32)	88 (27)	71 (36)	35 (39)	
Regional clinic	99 (16)	64 (19)	20 (10)	15 (17)	
Hospital dentistry	98 (16)	55 (17)	31 (16)	12 (13)	
**The use of a questionnaire**					0.004
Yes	60 (9.8)	23 (7.1)	20 (10)	17 (19)	
No	550 (90)	302 (93)	175 (90)	73 (81)	
(Missing)	4	4	0	0	
**Actual criteria for diagnosing dental phobia in new patients**					
Patient's complaining of anxiety	546 (89)	287 (87)	177 (91)	82 (91)	0.400
Experience of feeling unwell during treatment	397 (65)	206 (63)	131 (67)	60 (67)	0.500
Difficulty in keeping the patient's mouth open during treatment	174 (28)	101 (31)	38 (19)	35 (39)	0.001
Body movement during treatment	391 (64)	229 (70)	108 (55)	54 (60)	0.003
Cancellation of dental reception	149 (24)	80 (24)	46 (24)	23 (26)	> 0.9
**Need for establishment of standardized diagnostic method**					0.004
Very needed/a little needed	446 (73)	257 (78)	127 (65)	62 (69)	
Either/not needed a little/no need at all	168 (27)	72 (22)	68 (35)	28 (31)	
**Method for management**					
Behavioral therapy	384 (63)	264 (80)	66 (34)	54 (60)	< 0.001
Intravenous sedation	412 (67)	162 (49)	170 (87)	80 (89)	< 0.001
General anesthesia	283 (46)	125 (38)	98 (50)	60 (67)	< 0.001
Nitrous oxide inhalation sedation	296 (48)	165 (50)	80 (41)	51 (57)	0.028
Oral sedation	55 (9.0)	17 (5.2)	23 (12)	15 (17)	< 0.001
Others	25 (4.1)	17 (5.2)	6 (3.1)	2 (2.2)	0.400
(Missing)	1	0	1	0	

JSDH = Japanese Society for Disability and Oral Health, JDSA = Japanese Dental Society of Anesthesiology

^*^Pearson's Chi-squared test

Only 9.8% (60/614) of the participants used a questionnaire to diagnose dental phobia. The members of the both JSDH and JDSA group used questionnaires more frequently than members of the JSDH only group (17/90 [19%] and 23/329 [7.1%], respectively; corrected p < 0.01). Eighty-nine percent (546/614) of participants diagnosed dental phobia based on patient complaints of anxiety, and 65% (393/614) by experience of feeling unwell during treatment. Difficulty in keeping the patient's mouth open during treatment was used more frequently as a diagnostic criterion among the JSDH only group members than among the JDSA only group members (101/329 [31%] and 38/195 [19%], respectively; corrected p = 0.02). Body movement during treatment was also used more commonly as a diagnostic criterion among the JSDH only group members than among the JDSA only group members (229/329 [70%] and 108/195 [55%], respectively; corrected p < 0.01).

Table 3 shows the various dental anxiety assessment questionnaires used by practitioners. Sixty-four percent of the respondents (39/60) who used questionnaires used the original evaluation criteria for the diagnosis of dental anxiety.

**Table 3:** Questionnaires used by respondents

Questionnaires	n (%)
Modified Dental Anxiety Scale	9 (15.1)
Dental Fear Survey	6 (10.3)
The short version of the Dental Anxiety Inventory	2 (2.4)
Dental Subscale of Children's Fear Survey Schedule	1 (1.6)
Visual Analog Scale	12 (19.8)
State-Trait Anxiety Inventory	4 (6.3)
State-Trait Anxiety Inventory for Children	1 (1.6)
Original evaluation criteria	39 (64.3)

Respondents were able to choose more than one

Seventy-three percent of respondents (446/612) felt that an established standardized diagnostic method for dental phobia was needed accordingly (Table 2). A greater number of members of the JSDH only group felt the need for standardized diagnostic methods than those of the JDSA only group (257/329 [78%] and 127/195 [65%], respectively; corrected p < 0.01).

### Multiple regression analysis

Table 4 shows the associations between the use of dental anxiety assessment questionnaires and participants’ characteristics. Eleven participants were excluded due to missing data on the variables used, and 603 participants were hence finally included in the analysis. The factors that found a statistically significant association with the use of dental anxiety assessment questionnaires were membership of the JSDH only group (OR 0.28, 95% CI 0.13–0.60, p = 0.001) compared to both JSDH and JDSA and use of behavioral therapy (OR 2.34, 95% CI 1.18–4.84, p = 0.018).

**Table 4:** Results of a logistic regression analysis for the usage of questionnaire to diagnose dental phobia

Variables	OR	95% CI	P
Gender			
Female	–	–	–
Male	1.78	0.98, 3.32	0.063
Memberships			
Both JSDH and JDSA	–	–	–
JSDH only	0.28	0.13, 0.60	0.001
JDSA only	0.63	0.30, 1.35	0.23
Specialist	0.60	0.29, 1.20	0.16
Affiliated facility type	1.34	0.67, 2.74	0.42
Behavioral therapy	2.34	1.18, 4.84	0.018
Intravenous sedation	1.41	0.66, 3.10	0.38
General anesthesia	1.24	0.64, 2.49	0.53
Nitrous oxide inhalation sedation	0.94	0.52, 1.71	0.85
Oral sedation	0.91	0.34, 2.15	0.84

OR = Odds Ratio, CI = Confidence Interval, JSDH = Japanese Society for Disability and Oral Health, JDSA = Japanese Dental Society of Anesthesiology

## Discussion

This study investigated the methods of diagnosis of dental phobia using an online survey among Japanese dental practitioners who specialize in special-needs dentistry and dental anesthesiology. The results showed that the use of questionnaires was very low, patients’ subjective opinions were mostly used to diagnose dental phobia, and establishment of standardized diagnostic criteria was needed among practitioners. These results supported our hypothesis.

Only 9.7% of the practitioners included in the analysis who specialized in special-needs dentistry and dental anesthesiology used questionnaires to quantify the level of dental anxiety. These findings can be compared with those of the UK- and Australia-based studies. Among practitioners interested in behavioral sciences in the UK, 20% used questionnaires [21], and 3.4% of general dentists in Australia used published scales [22]. The difference between the results of our study and those of these studies could be explained by the differences in the composition of the study population. Nonetheless, the use of questionnaires for the diagnosis of dental anxiety is uncommon among dentists in Japan.

In this study, we observed that almost 90% of the participants diagnosed dental phobia based on complaints of fear of dental treatment, which is a similar finding to that of a previous study conducted in Australia, which showed that almost one-half of dentists directly asked their patients about dental anxiety [22]. Several standardized methods for the objective and quantitative evaluation of dental phobia are clinically applied; however, dentists continue to diagnose dental phobia or high-level dental anxiety based on subjective and original methods.

The results of multivariate analysis showed that membership of the JSDH only group was negatively related to the use of a questionnaire compared to the both JSDH and JDSA group. In addition, we found that members of the JSDH only group were more likely to diagnose dental phobia by the patient's body movements than other groups, and that they also had a greater need to establish standardized diagnostic criteria. There are two possible reasons why the JSDH only group members did not use the questionnaire. First, as mentioned above, the concept of measuring dental anxiety is not common in Japan. This is because it is not part of the model core curriculum, which is the government's summary of the minimum educational content before graduation [23]. Therefore, dentists may not know that a questionnaire has been developed for this purpose. Another reason for this is the patient population.

Members of the JSDH only group would treat more patients with disabilities combined with dental phobia than those with dental phobia but no disability. Many of the questionnaires that have been developed are self-administered; therefore, they are difficult to apply to patients with severe disabilities. A study using a validated questionnaire to measure dental fear in patients with mild to moderate intellectual disability reported that patients with a higher degree of intellectual disability had a higher level of dental fear [24]. The results of this study may suggest that the development of new diagnostic methods for dental fear in patients with disabilities is needed among dentists who specialize in special-needs dentistry. This study did not investigate the reasons why the participants did not use the existing questionnaire. Further investigation is needed to determine why they are not used and what diagnostic criteria are needed.

In contrast, these results also revealed that the use of behavioral therapy was positively related to the use of a questionnaire. Behavioral therapy is a psychological approach for the management of dental phobia [2]; therefore, analysis of dental anxiety using published tools would be common among dentists who use behavioral therapy. A previous study reported that female dentists and dentists who used the sedation method tended to use questionnaires more frequently [21]. However, these tendencies were not observed in the present study. Further research using larger samples is required accordingly.

The presence or absence of a specialist qualification (the required years of experience = 5 years) was not associated with the use of the questionnaire. In Australia, younger dentists are more likely to have received education related to the diagnosis and treatment of dental phobia and to report greater concerns about dental anxiety [22]. Age as a factor was not included in this study; however, it is expected that members who have a specialization are older than those who do not. Furthermore, in Japan, education on dental anxiety seems to be inadequate because the model core curriculum does not include items related to dental anxiety and dental phobia [23]. These factors would suggest that the age of dentists was not related to their interest in dental phobia in Japan.

The main limitation of this study is the low response proportion (7.8%); therefore, our results may not be generalizable to all members of the JDSA and JSDH. It is possible that the members who participated in the study were more likely to have an interest in dental phobia than those who did not participate. The target population may have used fewer questionnaires and may have had a lesser need for a standardized diagnostic method for dental phobia. The number of subjects using the questionnaire was also small (60 participants). Therefore, the effect of random errors may be present in factors related to the use of the questionnaire. To overcome this issue, it is necessary to conduct a large-scale survey. However, this is the first study to assess the methods of diagnosis for dental phobia used by dentists who specialize in special-needs dentistry and dental anesthesiology.

## Conclusions

In our study population, the use of questionnaires was very low, patients’ subjective opinions were commonly used to diagnose dental phobia, and establishment of standardized diagnostic criteria were needed among the practitioners. Therefore, it is necessary to establish diagnostic criteria in line with the Japanese clinical system for dental phobia and to educate dentists about the criteria.

## Data Availability

The datasets used and/or analyzed during the current study are available from the corresponding author upon reasonable request.

## Abbreviations

CBT: Cognitive Behavioral Therapy
DSM-5: Diagnostic and Statistical Manual of Mental Disorders, Fifth Edition
JSDH: Japanese Society for Disability and Oral Health
JDSA: Japanese Dental Society of Anesthesiology
